# Durability assessment results suggest a serviceable life of two, rather than three, years for the current long-lasting insecticidal (mosquito) net (LLIN) intervention in Benin

**DOI:** 10.1186/1471-2334-14-69

**Published:** 2014-02-08

**Authors:** Virgile Gnanguenon, Roseric Azondekon, Frederic Oke-Agbo, Raymond Beach, Martin Akogbeto

**Affiliations:** 1 Centre de Recherche Entomologique de Cotonou (CREC), Cotonou, Benin; 2 Faculte des Sciences et Techniques de l’Université d’Abomey-Calavi, Abomey-Calavi, Benin; 3 University of Massachusetts, Amherst, MA, USA; 4 Centers for Disease Control and Prevention, Atlanta, GA, USA

**Keywords:** Long-lasting Insecticidal Net (LLIN), Survivorship, Fabric integrity, LLIN intervention serviceable life, Durability

## Abstract

**Background:**

LLIN distribution, every three years, is a key intervention of Benin’s malaria control strategy. However, data from the field indicate that LLIN lifespan appears to vary based on both intrinsic (to the LLIN) and extrinsic factors.

**Methods:**

We monitored two indicators of LLIN durability, survivorship and integrity, to validate the three-year-serviceable-life assumption. Interviews with net owners were used to identify factors associated with loss of integrity.

**Results:**

Observed survivorship, after 18 months, was significantly less (p<0.0001) than predicted, based on the assumption that nets last three years. Instead, it was closer to predicted survivorship based on a two-year LLIN serviceable life assumption (p=0.03). Furthermore, the integrity of nearly one third of ‘surviving’ nets was so degraded that they were in need of replacement. Five factors: washing frequency, proximity to water for washing, location of kitchen, type of cooking fuel, and low net maintenance were associated with loss of fabric integrity.

**Conclusion:**

A two-year serviceable life for the current LLIN intervention in Benin would be a more realistic program assumption.

## Background

National distribution of long-lasting insecticidal (mosquito) nets (LLINs) is a proven malaria control intervention [1-3]. However, LLIN interventions have a limited serviceable life, and net replacement must be programmed in a timely way to maintain impact. Programs that wait too long risk operational failure; likewise, premature replacement is to be avoided for cost-effectiveness reasons. The current assumption regarding LLIN serviceable life is three to five years of use under field conditions [4,5]. While this has been incorporated into LLIN intervention planning, questions have arisen about whether such an approach holds true everywhere nets are in use [6]. Rather than a single LLIN serviceable life everywhere, data from the field indicate that LLIN loss appears to vary based on both intrinsic (to the LLIN) and extrinsic factors [7-10]. We monitored LLIN durability in Benin, during the current national LLIN distribution – replacement cycle, so that the timing of the future replacement campaigns can be informed by Benin-specific net loss data.

## Methods

**Figure 1:**
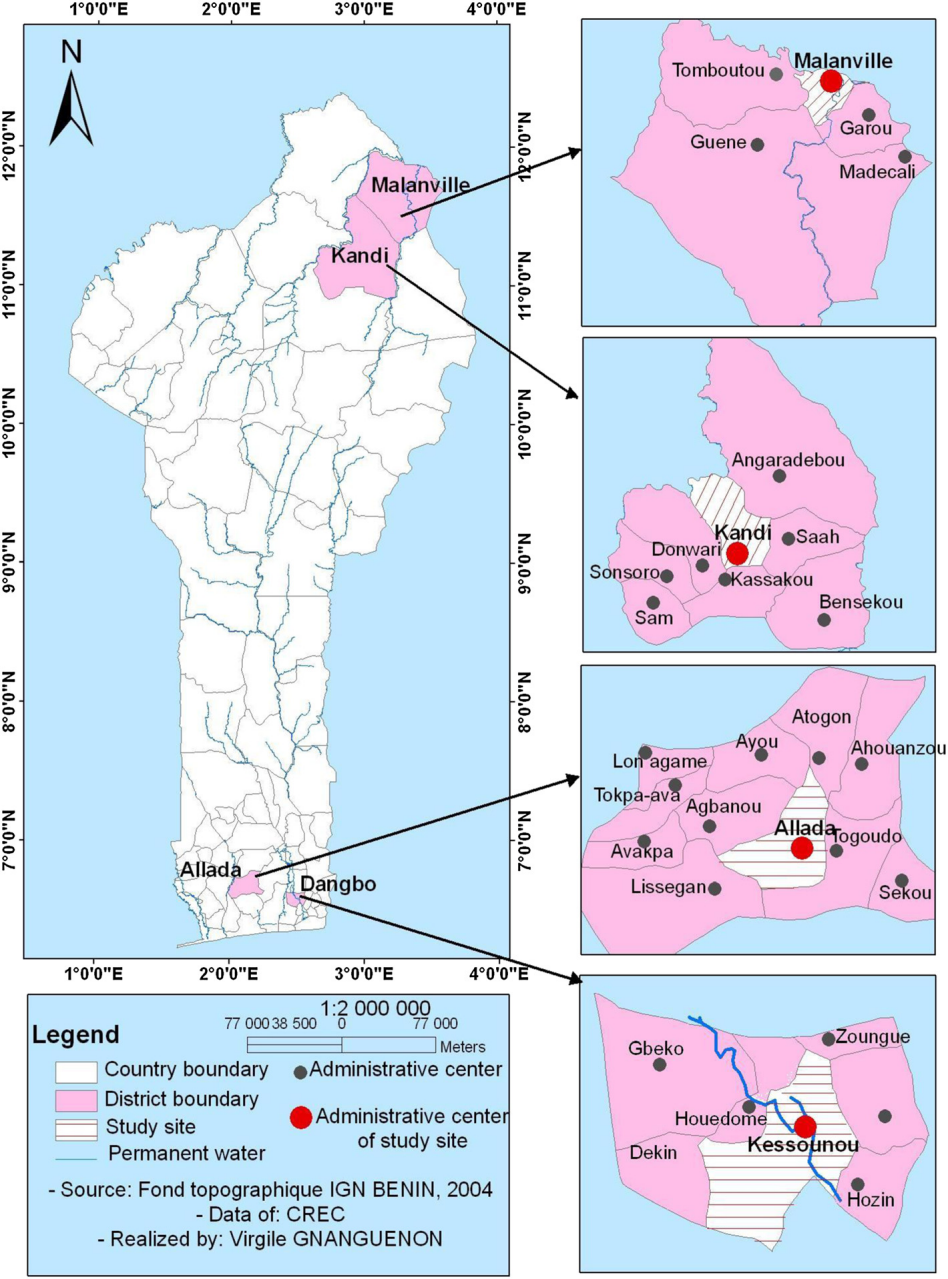
**Reproduced copy (with copyright) of Benin Map LLIN tracking assessment sites: Kessounou, Allada, Kandi and Malanville **[11]

Beginning in July, 2011, approximately four million polyethylene-based LLINs (Olyset® net, Sumitomo Chemical Company) were distributed throughout Benin. Following distribution, a net tracking activity to monitor the durability of the LLINs [11,12] was implemented in four communities. Two of the communities, Kessounou and Allada, were located in Southern Benin (Figure 1), while the other two, Kandi and Malanville, were located approximately 750 km further North. Residents in Kessounou, located on the Oueme River, have ready access to water for washing nets. In contrast, residents of Allada, who want to wash a net, must carry water some five kilometers. Similar criteria (a short distance to water for washing LLINs versus a long distance) applied to the sites in the North. Malanville, located on the Niger River (water for washing nets easily accessible) and Kandi (residents must transport water for washing nets, as at Allada).

As described previously, at the time of the nationwide LLIN distribution in 2011, a sample of 2002 households, approximately 500 per site, was randomly selected [11]. Household selection at each site took into account all villages to ensure representative sampling [11]. Assessment teams identified a 2011-distribution LLIN in each selected household and, if the net was hanging and in use, enrolled it, and the household where it was located, in a LLIN durability monitoring assessment, based on World Health Organization guidelines [13]. Each selected LLIN was double tagged (a bar code, attached to the net, plus an indelible-ink symbol applied to the LLIN fabric) to ensure correct identification during later visits. The GPS coordinates of the household and the name of the head of household, or an adult person acting on behalf of the head, were also recorded to facilitate follow up. This paper reported the 12 and 18 months assessments.

### Monitoring survivorship/Attrition

Due to the way in which households were selected (one enrolled LLIN per household), LLIN survivorship at T_0_, was set at 100%. At 6-month intervals, each selected household received a follow-up visit. If the household was open at the time of the visit, the assessment team visually confirmed the continued presence of the coded LLIN. If the coded LLIN was not in the house, the assessment teams determined how the net went missing by interviewing the owner. Owners were asked to choose one of three reasons for why the net was no longer present in the household. These were: (i) the net was thrown away because it was physically damaged and thought to be of no value, (ii) the net was removed (e.g. given away, stolen, sold etc.), and (iii) the net was re-purposed for an alternative use.

### Monitoring integrity

LLIN fabric integrity was assessed by a visual examination, without removal of coded nets from selected households. Observed holes were assigned to one of four size categories:

1. a hole size of 0.5-2.0 cm or ‘<a thumb-sized opening’

2. a hole size of 2.0-10.0 cm or ‘>a thumb but<a fist’

3. a ‘hole size of 10–25 cm or ’>a fist but<a head’

4. a hole size of >25 cm or ‘>a head’

The most-likely cause of the damage, a rip in the fabric, a rip in the seam, burned burn-related hole or the result of rodent damage was also recorded.

### Interview questionnaire

A questionnaire, developed by WHO [13], to identify factors associated with survivorship, was adapted for use in the assessment. Questions were programmed (ODK Collect 1.2.2 software) into Samsung Galaxy Tablets to record responses.

### Data analysis

#### Survivorship

The equation for quantifying overall survivorship, also referred to as attrition, was:

$$\frac{\mathrm{Total}\;\mathrm{coded}\;\mathrm{LLINs}\;\mathrm{still}\;\mathrm{present}\;\mathrm{in}\;\mathrm{the}\;\mathrm{households}\;\mathrm{selected}\;}{\mathrm{Total}\;\mathrm{coded}\;\mathrm{LLINs}\;\mathrm{at}\;\mathrm{enrollment}\;\left(T_{0}\right)}\times 100$$

If a household was closed, during an assessment visit, it was treated according to the non-parametric survival method of Kaplan-Meier [14]. Survivorship, plotted against time (T_6_, T_12,_ T_18),_ was compared with NetCALC net loss model curves based on 2-year and 3-year LLIN serviceable life assumptions (http://www.networksmalaria.org). Equations for calculation of LLIN survivorship/attrition associated with three different reasons for why an assessment net had gone missing were:

Attrition rate-1 (reason: physical damage):

$$\begin{array}{l} \text{Total number of coded LLIN reported as thrown out}\;\\ \;\frac{\text{due to wear and tear in surveyed households}\;}{\text{Total coded LLINs at enrollment}\;\left(T_{0}\right)}\times 100 \end{array}$$

Attrition rate-2 (reason: removal):

$$\begin{array}{l} \text{Total number of coded LLIN reported as given away},\\ \;\frac{\text{stolen},\;\text{sold or used in another location}\;}{\text{Total coded LLINs at enrollment}\;\left(T_{0}\right)}\times 100 \end{array}$$

Attrition rate-3 (reason: re-purposed):

$$\begin{array}{l} \text{Total number of coded LLIN reported as being used}\\ \;\frac{\text{for another purpose in surveyed households}\;}{\text{Total coded LLINs at enrollment}\;\left(T_{0}\right)}\times 100 \end{array}$$

Two communities were reported to show significantly different survivorship/attrition if the 95% confidence limits did not overlap.

*Integrity* was quantified based on two measurements:

1) The proportion of LLINs with any hole.

$$\begin{array}{c} \text{Total number of coded LLINs with}\\ \;\frac{\text{at least one hole of size}\;1‒\;4}{\text{Total number of coded LLINs found and}\;}\times 100\\ \;\text{assessed in surveyed households} \end{array}$$

2) The proportionate holes index (pHI) for each net [13]

1 × *number of size* − 1 *holes* + 23 × *no. of size* − 2 *holes* + 196 × *no. of size* − 3 *holes* + 576 × *no. of size* − 4 *holes*. The figures, 23, 146, and 576 refer to the estimated mean hole area for the different sized holes. Descriptive statistics were used to compare pHI values at each assessment site (mean, median, interquartile range). Based on the pHI score, LLINs were assigned to one of three condition catagories (Roll Back Malaria: Measurement of Net Durability in the Field: Current Recommended Methodology, presented in Lyon, February 2012).

*pHI ≤64 -****good***

*pHI ≤768 -****serviceable***

*pHI >768 -****replace***

Factors associated with loss of integrity were identified by multivariate regression analysis of nets in the ‘replace’ category and frequency of responses (by owners of the nets). Modalities with very low numbers observed were aggregated with those that have high numbers for the multivariate analysis.

### Study clearance

This prospective study was planned with and approved by the Ministry of Health. Community leaders were informed before the study and all gave verbal consent before initiation. Written consent was then obtained on the day of the study from all participating households.

## Results

### Net survivorship/attrition

There were 2002 nets enrolled (T_0_) in the assessment. During the T_6_ T_12_ and T_18_ follow up visits, 1672, 1225, and 973 LLINs, respectively, were found and evaluated (Table 1). There was a significant difference in survivorship associated with community location, but not associated with distance to water for washing nets. After 12 months, the estimated survivorship in the South 65% [CI 95%: 62.54-68.41], was significantly lower than in the North, 78% [CI 95%: 75.04-80.19]. In contrast, when survivorship data were analyzed by ease of access to water for washing nets, no significant differences were observed. After 12 months it was 71% [CI 95%: 67.87-73.49] for the ‘distant’ (from water) communities and 73% [CI 95%: 70.06-75.56] for the ‘near’ (to water) ones. The same pattern was observed after 18 months. Estimated mean survivorship was 50% [CI 95%: 46.86-53.04] in the South, significantly lower than in the North, 65% [CI 95%: 61.83-67.73]; and 57% [54.00-60.12] for the ‘distant’ (from water) communities versus 58% [CI 95%: 54.61-60.73] for the ‘near’ (to water) ones. In summary, survivorship (all communities) was 93% after 6 months, 72% after 12 months and 57% after 18 months; was variable between southern and northern localities; but did not appear to change based on distance to water for washing (LLINs loss in South was 01.62 [CI 95%: 01.41-01.86] times that in the North, but the same at sites near water versus those located farther away.

**Table 1:** LLIN survivorship by assessment community

**Water for washing nets**		**Kessounou (South)**	**Malanville (North)**	**Allada (South)**	**Kandi (North)**	**Total**
		**Near**		**Distant**		
Distance to water for washing LLINs		<0.05 km	<0.05 km	>5.0 km	>5.0 km	
Baseline (T_0_)	Households enrolled	501	501	500	500	2002
After 6 months (T_6_)	Households eligible	501	501	500	500	2002
	Households visited/opened	493	455	420	451	1819
	Coded LLINs found	444	424	374	430	1672
	(LLINs lost)	(49)	(31)	(46)	(21)	(147)
	Survivorship (%)	90	94	91	96	93
After 12 months (T_12_)	Households eligible	452	470	454	479	1855
	Households visited/opened	393	411	396	442	1642
	Coded LLINs found	253	338	286	348	1225
	(LLINs lost)	(140)	(73)	(110)	(94)	(417)
	Survivorship (%)	62	79	69	77	72
	95% confidence interval	58.20-66.70	75.80-82.90	64.90-73.00	73.40-80.80	69.90-73.80
After 18 months (T_18_)	Households eligible	312	397	344	385	1438
	Households visited/opened	293	350	274	345	1262
	Coded LLINs found	227	279	184	283	973
	(LLINs lost)	(66)	(71)	(90)	(62)	(289)
	Survivorship (%)	49	65	51	65	57
	95% confidence interval	44.90-53.70	62.90-71.10	46.60-55.40	58.70-67.20	55.30-59.60

**Figure 2:**
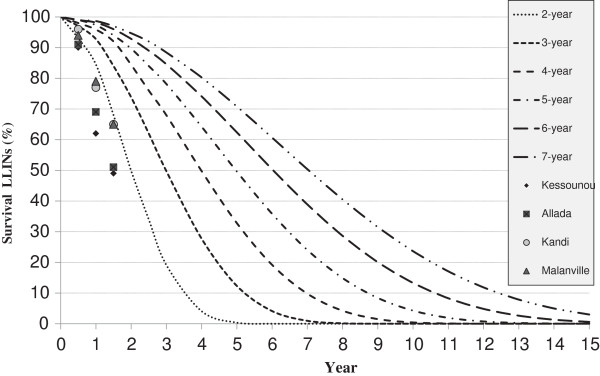
Estimated LLIN survival at 6, 12 and 18 months in four communities compared with NetCALC* 2-year, 3-year, 4-year, 5-year, 6-year and 7-year net loss model curves.

Observed survivorship is compared with NetCALC loss predictions (NetCALC loss curves) in Figure 2. NetCALC predicted survivorship at 18 months is 84% for the three year model, and 68% for the 2-year model, whereas observed survivorship in all communities at 18 months, 57%, is significantly less (P<0.0001) than predicted based on the three-year assumption and also less than, but closer to, that predicted by the NetCALC 2-year loss rate model (p=0.036).

### Reasons for net loss

There were 313 and 289 interviews, at households where the net was missing, administered during the 12 and 18 month follows up visits. ‘Removal’, the most commonly cited reason for a missing net, was mentioned in 240 (range 34–85, by site) and 154 (range 26–59, by site) of the interviews at 12 and 18 months respectively (Table 2). ‘Net thrown away due to physical damage’ was cited 118 (range 7–79, by site) and 132 (range 27–38, by site) times at T12 and T18 respectively. ‘Repurposing’ was mentioned least often, 59 (range 5–27) and 3 (range 0–2) at T12 and T18 respectively. Estimated LLIN attrition, associated with ‘net thrown away’ from T_0_ to T_12_ was 7% (range 2-18%) for, for net ‘removed’, 18% (range 14-25%), and for ‘net re-purposed, 3% (1-6%). After 18 months, the estimated attrition rates by reason were 14% (range 7-25%) for ‘net thrown away’, 26% (range 19-37%) for ‘net removed’ and 3% (1-7%) for ‘net re-purposed’. Physical damage’ was cited more often in the communities where nets were washed more often, e.g. communities located *near* water for washing nets, 19% of responses [CI 95%: 16.18-20.98] in Malanville and Kessounou, versus 9% [CI 95%: 06.84-10.28] in Allada and Kandi. “Removal,’ the most common fate of a missing LLIN, was cited more often in the communities distant from water for washing nets 31% [CI 95%: 28.40-34.14] versus 21% [CI 95%: 18.17-23.17].

**Table 2:** **Reasons for net loss (attrition): T**_**12 **_**and T**_**18 **_questionnaire* response summary

		**Kessounou (South)**	**Malanville (North)**	**Allada (South)**	**Kandi (North)**	**Total**
After 6 months (T_6_)	(LLINs lost)	(49)	(31)	(46)	(21)	(147)
‘physical damage’ responses		10	3	5	1	19
‘removal’ responses		35	28	41	20	124
‘re-purposed’ responses		4	0	0	0	4
After 12 months (T_12_)	(LLINs lost)	(140)	(73)	(110)	(94)	(417)
Questionnaires administered		140	73	110	94	417
‘physical damage’ responses		79	19	13	7	118
‘removal” resonses		34	49	85	72	240
‘re-purposed’ responses		27	05	12	15	59
Attrition rate-1 (%)		18	05	04	02	07
95% confidence interval		14.67-21-35	02.92-06.56	02.29-05.62	00.81-03.13	05.82-08.03
Attrition rate-2		14	15	25	18	18
95% confidence interval		11.03-17.07	12.48-18.79	21.59-29.18	15.25-22.03	16.55-19.93
Attrition rate-3		06	01	02	03	03
95% confidence interval		04.-39-08.65	00.43-02.31	01.38-04.15	01.83-04.89	02.47-04.01
% net loss (total attrition)		38	21	31	23	28
After 18 months (T_18_)	(LLINs lost)	(66)	(71)	(90)	(62)	(289)
Questionnaires administered		66	71	90	62	289
‘physical damage’ responses		38	36	31	27	132
‘removal’ responses		26	34	59	35	154
‘re-purposed’ responses		2	1	0	0	3
Attrition rate-1		25	12	10	07	14
95% confidence interval		21.74-29.34	09.06-14.68	07.49-12.72	05.08-09.58	12.01-15.00
Attrition rate-2		19	22	37	25	26
95% confidence interval		15.77-22.63	18.74-26.00	32.88-41.32	21.78-29.39	24.00-27.84
Attrition rate-3		07	01	02	03	03
95% confidence interval		04.73-09.11	00.55-02.59	01.38-04.15	01.83-04.89	02.60-04.17
% net loss (total attrition)		51	35	49	35	43

*administered to all households if open and missing coded net.

### LLIN fabric integrity

Of the nets remaining in the households to which they were distributed, the percentage with any hole, ranged from 62% at Allada to 87% at Malanville and Kessounou at T12 (Table 3). At T18, the percentage of LLINs with any hole, ranged from 72% at Allada to 93% at Malanville. Consistent differences in measures of integrity were observed when results for the two communities that were near the source of water for washing nets were compared with the two communities that were farther away from the source of water for washing (Table 3). By the end of one year (T12), the mean proportionate hole indices in the communities located near water for washing (area of greater washing frequency) were 691 and 799, versus 398 to 510 in the communities located farther from washing (lower LLIN washing frequency). Additionally, median pHI values for the communities nearer to the ‘wash water’ source, 290 and 243, were three to six times greater than that observed in communities that were farther away from water for washing nets, 86 and 46. Applying the net condition categories, ‘good’, ‘serviceable’ and ‘needs replacement’, 33 and 30 percent of the nets in communities near water for washing were in need of replacement versus 16 and 22 percent in the communities that were farther away from water. Furthermore, the number of LLINs in the ‘good condition’ category was significantly higher (p<0.05) in location with less access to water. At the 18-month assessment visit, the mean proportionate hole indices in the communities located near water for washing (area of greater washing frequency) were 888 and 1479, versus 623 to 447 in the communities located farther from washing (lower LLIN washing frequency). Median pHI values for the communities nearer to a ‘wash water’ source, 706 and 312, were also greater than that observed in communities that were farther away from water for washing nets, (105 and 96). There were 48 and 36 percent of the nets in communities near water for washing in need of replacement, versus 24 and 18 percent in the communities that were farther away from water (Table 3). LLINs in the ‘good condition’ category were also significantly higher (p<0.05) in location with less access to water for washing nets. When LLINs with any holes were categorized by the nature of hole as representing either a rip in the fabric, a rip in the seam, a burn hole or the result of chewing by rodents, the ‘rip in the fabric’ category accounted in average for 85% of the damage, while burn holes accounted for 10%, open seams for 3% and rodent damage for 2% at T12 assessment (Table 4). At T18 assessment, the ‘rip in the fabric’ category accounted in average for 84% of the damage, while burn holes accounted for 11%, open seams for 3% and rodent damage for 2% (Table 4).

**Table 3:** LLIN Fabric integrity (pHI) after 12 and 18 months at sites with different access to water for washing nets

	**Kessounou South**		**Malanville North**		**Allada South**		**Kandi North**	
	**T12**	**T18**	**T12**	**T18**	**T12**	**T18**	**T12**	**T18**
Tagged LLINs found	253	227	338	279	286	184	348	283
n (%) of nets found with any hole (s)	219 (87)	210 (92)	294 (87)	260 (93)	178 (62)	133 (72)	241 (69)	208 (73)
CI_95_ of (%)	81.73-90.51	88.28-95.58	82.92-90.38	89.57-95.85	56.34-67.88	65.22-78.62	64.11-74.06	67.95-78.55
Mean pHI	799	1479	691	888	398	623	510	447
Median pHI	290	706	243	312	46	105	86	96
IQR pHI	1034	2057	978	1156	439	667	560	477
n (%) of nets in pHI<64 ‘good’ catagory	69 (27)	43 (19)	107(32)	73 (26)	162 (57)	83 (46)	161 (46)	133 (47)
CI_95_ (%)	22.16-33.07	14.38-24.54	26.93-36.80	21.36-31.62	50.8-62.26	39.2-53.67	41.10-51.52	41.26-52.81
n (%) of nets in 64<pHI<768 ‘serviceable’ catagory	100 (40)	74 (33)	129 (38)	105 (38)	79 (28)	53 (30)	112(32)	99 (35)
CI_95_	33.70-45.66	26.83-38.94	33.15-43.45	32.1-43.45	22.76-33.08	23.4-36.67	27.49-37.26	29.66-40.71
n (%) of nets in pHI >768 ‘needs replacement’ catagory	84 (33)	110 (48)	102 (30)	101 (36)	45 (16)	43 (24)	75 (22)	51 (18)
CI_95_	27.69-39.22	42.04-54.93	25.53-35.27	30.7-41.27	11.9-20.40	18.3-30.78	17.5-26.17	13.98-22.92

IQR=Interquartile range; n=number; T12=12 months; T18=18 months; CI=Confidence Interval.

**Table 4:** Type of damage: percentage of assessment households responding to LLIN survey question “What was the principle cause of the damage to this LLIN?” at 12 and 18 months assessment visits

	**Kessounou South**		**Malanville North**		**Allada South**		**Kandi North**	
Distance to water for washing nets	< 0.05 km				> 5.0 km			
Assessment visit	T12	T18	T12	T18	T12	T18	T12	T18
n of nets found with any hole (s)	219	210	294	260	178	133	241	208
n (%) of nets with ‘rip in the fabric’	158 (72)	142 (68)	279 (95)	248 (95)	133 (75)	114 (86)	219 (91)	178 (86)
CI 95%	65.87-77.66	61.02-73.58	91.75-96.88	92.11-97.34	67.86-80.54	78.76-90.66	86.57-93.89	80.16-89.71
n (%) of nets with ‘rip in the seam’	4 (2)	5 (2)	3 (1)	5 (2)	10 (6)	3 (2)	9 (4)	16 (8)
CI 95%	00.71-04.60	01.02-05.45	00.35-02.96	00.82-04.42	03.08-10.03	00.77-06.42	01.98-06.94	04.79-12.13
n (%) of nets with ‘burn holes’	52 (24)	62 (29)	9 (3)	6 (2)	26 (14)	11 (8)	12 (5)	9 (4)
CI 95%	18.59-29.80	23.77-36.02	01.62-05.71	01.06-04.94	10.17-20.54	04.68-14.20	02.87-08.50	02.29-08.02
n (%) of nets chewing by rodent	5 (2)	2 (1)	3 (1)	1 (1)	9 (5)	5 (4)	1 (0)	5 (2)
CI 95%	00.98-05.23	00.26-03.41	00.35-02.96	00.07-02.15	02.68-09.33	01.62-08.50	00.07-02.31	01.03-05.50

CI=Confidence Interval.

### Factors associated with loss of integrity

Table 5 characterizes households by LLIN washing frequency, nightly LLINs use, maintenance, and five other ‘selected’ characteristics, roofing material, daytime placement, kitchen location, cooking fuel, and type of sleeping furniture (bed, mat, other). Washing frequency increased more rapidly over time in communities that were closer to the water-for- washing source. Most households indicated that LLINs were used nightly (55-84%). Over half of the households use steel sheet as their roofing material. Most, 62-98%, used firewood for cooking fuel, mats were more common (50-95%) at Kessounou and Malanville, whereas, at Allada and Kandi beds were more common (44-51%).

**Table 5:** Percentage distribution of washing frequency, LLINs usage and housing characteristic by assessment visit (T12, T18)

**Factors**	**Modalities**	**KESSOUNOU**		**MALANVILLE**		**ALLADA**		**KANDI**	
		**T12**	**T18**	**T12**	**T18**	**T12**	**T18**	**T12**	**T18**
**Washing frequency**	None	08.16	02.17	04.00	00.00	28.00	31.91	14.29	22.45
	1 time	04.08	02.17	06.00	06.12	30.00	10.64	16.33	12.24
	2-5 times	61.22	36.96	52.00	24.49	38.00	44.68	63.27	48.98
	6-10 times	16.33	39.13	24.00	34.69	04.00	12.77	06.12	16.33
	10 and more	10.20	19.57	14.00	34.69	00.00	00.00	00.00	00.00
**LLINs maintenance**	Clean	30.61	41.30	46.00	26.53	28.00	25.53	36.73	28.57
	Dirty	69.39	58.70	54.00	73.47	72.00	74.47	63.27	71.43
**LLINs use**	Not at all	04.08	04.35	00.00	06.12	08.00	00.00	02.04	2.04
	Often	26.53	30.43	16.00	34.69	22.00	25.53	16.33	42.86
	Every night	69.39	65.22	84.00	57.14	70.00	74.47	81.63	55.10
**Roofing material**	Paving stone	2.04	02.17	02.00	01.15	02.00	02.13	01.00	02.04
	Straw	20.41	34.78	22.00	21.30	00.00	00.00	06.12	02.04
	Steel sheet	77.55	63.04	76.00	77.55	98.0	97.87	92.88	95.92
**Daytime location of LLIN**	Hanging	71.43	71.74	74.00	59.18	64.00	80.85	81.63	71.43
	Folded	22.45	23.91	26.00	36.73	20.00	14.89	16.33	28.57
	Tidy away	6.12	04.35	00.00	04.08	16.00	04.26	02.04	00.00
**Location of the kitchen**	Outside	83.67	76.52	90.00	100.0	96.00	93.62	97.96	100.0
	Inside	16.33	23.48	10.00	00.00	04.00	06.38	02.04	00.00
**Cooking fuel**	Firewood	97.96	97.83	90.00	97.96	62.00	68.09	67.35	87.76
	Chacoral	02.04	02.17	08.00	02.04	36.00	29.79	30.61	10.20
	Gas	00.00	00.00	02.00	00.00	02.00	00.00	02.04	02.04
	kerosene	0.00	00.00	0.00	00.00	0.00	02.13	0.00	00.00
**Sleeping material**	Other	0.00	00.00	8.00	12.24	4.00	02.13	20.41	10.20
	Bed	4.08	08.70	42.00	30.61	44.00	48.94	51.02	51.02
	Matting	95.92	91.30	50.00	57.14	52.00	48.94	28.57	38.78
**Total number of households visited/opened**		393	293	411	350	396	274	442	345

Factors that showed a significant relationship with loss of integrity as measured by nets with any hole included: washing frequency, LLINs maintenance (low), location of the kitchen (inside the house), type of cooking fuel and the distance to water for washing (Table 6). High washing frequency increased the risk of physical damage to the LLINs (p<0.0001).

**Table 6:** Factors associated with loss of integrity

**Factors**	**Modalities**	**Coefficients**	**Rate ratio**	**CI-% (RR)**	**P (Wald test)**	**P (LR-test)**
**Washing frequency**	None	-	1.00	-	-	<0.0001
	1	0.744	2.1	[0.92-04.84]	0.07967	
	2-5	1.024	2.78	[1.40-05.55]	0.00355	
	>=6	1.766	5.85	[2.85-11.99]	<0.0001	
**Type of roof**	Steel sheet	-	1.00	-	-	0.168491
	Straw	−0.324	0.72	[0.46-01.15]	0.16930	
**Location of kitchen in the house**	Outside	-	1.00	-	-	0.026399
	Inside	0.543	1.72	[1.06-02.79]	0.027699	
**Cooking fuel**	Charcoal	-	1.00	-	-	0.034210
	Firewood	0.582	1.79	[1.04-03.09]	0.036875	
**Sleeping material**	Other	-	1.00	-	-	0.774194
	Bed	−0.229	0.79	[0.43-01.49]	0.472834	
	Matting	−0.142	0.87	[0.48-01.56]	0.6365219	
**LLINs maintenance**	High	-	1.00	-	-	0.002677
	Low	0.526	1.69	[1.20-02.39]	0.002908	
**Frequency of net use**	Not at all	-	1.00	-	-	0.077341
	Often	−0.889	0.41	[0.19-00.88]	0.02139	
	Every night	−0.718	0.49	[0.24-01.00]	0.05074	
**Distance to water for washing**	>5 km	-	1	-	-	0.000106
	<0.5 km	0.48	1.61	[1.27-2.05]	0.000106	
**Number of sleeper/Net**	-	0.105	1.11	[0.96-01.28]	0.14466	0.153694

RR=Rate Ratio; P=p-value.

## Discussion

LLIN loss, measured by survivorship/attrition, occurred more rapidly than predicted by the ‘three-year serviceable life’ assumption, currently used in Benin to program distribution/replacement of LLINs. Of interest, however, was the observation that survivorship rates did not appear to be affected by LLIN washing frequency. In contrast, our results showed a marked effect of washing frequency on the integrity of the LLINs. Therefore, LLIN loss of physical integrity/deterioration, a factor that affects the ability of LLINs to prevent mosquito-human contact, may not be taken into account if only survivorship is monitored. In this evaluation, loss of integrity, measured in LLINs that remained in place, was so extensive that it may well have compromised the value of the LLINs still remaining in the households as a malaria prevention measures. Recent work [15] in which damaged LLINs are shown to increase man-vector contact from none to an average of five bites/man/night demonstrates the impact that a relatively small loss of integrity, increasing the pHI to 276, can have on LLIN efficacy. Therefore, we suggest that, assumptions about LLIN serviceable life/time to replacement should be informed, to the extent possible, by monitoring integrity of existing nets [13] as well as survival/attrition. Our results suggest that the assumption of a 3-year LLIN serviceable life, for the LLINs distributed in 2011 in Benin overestimated their duration of impact by as much as one year, a situation that could contribute to a rebound in malaria illness during the last year (year three) prior to net replacement. Additional programmatic questions of interest that can be addressed by LLIN durability monitoring include: ‘ Is the programmatic ‘effective life assumption realistic under local/regional conditions?’; ‘Do some LLIN products have slower loss/integrity attrition rates in a given location?’; and, ‘What programmatic changes can be implemented to improve LLIN duration of effective life?’

Net removal for all reasons was observed in 6% of the study houses at T6, in 18% at T12 and in 26% at T18. Some, perhaps most, of these nets were moved by design of the household (e.g. given away to other houses in the community) and therefore, it is possible that the nets continued to contribute to community protection. Nonetheless, the percentage of nets thrown away (25%) and nets re-purposed were surprisingly high in some communities, eg. Kessounou.

The proportionate hole index (pHI) [13] provides a standardized approach to describing changes in LLIN fabric integrity. Applying pHI thresholds [16]: ‘like new’, ‘needs repair’ and ‘needs replacement’, to our results, we observed that after 12 months, 16-22% of the LLIN need to be replaced at locations with less access to water (Allada and Kandi) versus 30-33% at locations with ready access to water for washing (Malanville and Kessounou). Estimates of net loss associated with integrity (computing loss as proportion of nets in pHI category ‘needs replacement’ were between 18% and 48% after 18months). If loss associated with fabric integrity declines at these rates, more than one half of the LLINs distributed in 2011, and still present in the household to which they were given, would need replacement prior to two transmission seasons post-distribution. Thus, based on fabric integrity alone, the LLINs would not be expected to provide adequate protection after two transmission seasons.

Cooking fuel, location of the kitchen as well as low LLINs maintenance and washing frequency were correlated with the loss of fabric integrity. Social practices regarding net care and repair, vary from community to community, and most likely influence condition of the LLINs in each region [17,18]. It important, therefore, to reinforce awareness about the best practices related to net care and repair.

A recent study in Kenya [19] reported that people washed their LLINs more frequently than recommended and associated this practice with poor physical quality of nets. The study also noted that light colored nets were more likely to be washed than were dark colored nets. The polyethylene-based LLINs in this assessment were light blue. The majority of the nets (54-74%) were observed to be dirty. Thus, it may be that these factors also contributed to the high observed frequency of washing/rapid decline in fabric integrity. They could significantly affect the bio-efficacy of the nets that decreased from 9-58% after 6 months [11]. However, bio-efficacy results were not included in the present assessment-and represent a potential limitation of the study.

The findings of this study have important implications for the LLIN-malaria control strategy in Benin. They suggest that: the current polyethylene-based LLIN, distributed during 2011, has an effective life of two rather than three years; that behaviour change communication (BCC) strategies that support LLINs repair and that discourage subsequent removal of nets after distribution should be strengthened in an attempt to sustain higher coverage levels for a longer period of time; and that channels for routine replacement of LLINs, between national campaigns should be strengthened to replace nets that no longer meet minimum fabric integrity standards; and that other WHOPES-approved LLIN products should be evaluated to determine which is most cost-effective. Finally, it may be that local communities can adopt and use WHO guidance on assessment of integrity (counting holes) and survival to assess LLIN loss, thereby verifying ongoing impact under local conditions.

## Conclusions

This study suggests that in Benin, survivorship/attrition of LLINs followed a 2-year net serviceable life assumption (model), rather than the currently used three-year assumption; that loss of fabric integrity in nets that were still in use, was extensive, with as many as one-third in need of replacement. The condition of the LLINs at the midpoint of its assumed serviceable life raises serious doubts about their usefulness in preventing mosquito-human contact during the subsequent 18 months prior to program replacement of nets under the current plan.

As countries consider how to sustain LLIN impact, it will be important to have meaningful information on LLIN loss in a variety of settings. This information should be used to ‘inform’ decisions on replacing LLINs thereby avoiding loss of efficacy earlier than assumed.

## Competing interests

The authors declare that they have no competing interests.

## Authors’ contributions

VG: designed experiments, coordinated field activities, mapping, collected data, wrote and revised the paper; RA: participated in designed experiments, field activities and revised the paper; FO: assisted with the statistical analysis and participated in field activities and data collection; RB: participated in the design of the study and revised the paper; AM: designed the study, supervised field activities and revised the paper. All authors have read and agreed with the content of the submitted manuscript.

## Pre-publication history

The pre-publication history for this paper can be accessed here:

http://www.biomedcentral.com/1471-2334/14/69/prepub
